# The preface: Interplay of topological and magnetic orders in the Mn-Bi-Te family

**DOI:** 10.1093/nsr/nwae024

**Published:** 2024-01-30

**Authors:** Fu-Chun Zhang, Hai-Zhou Lu, Xin-Cheng Xie

**Affiliations:** Kavli Institute for Theoretical Sciences, University of Chinese Academy of Sciences, China; Department of Physics, Southern University of Science and Technology (SUSTech), China; International Center for Quantum Materials, School of Physics, Peking University, China

The discovery and synthesis of MnBi_2_Te_4_ in 2013 initially garnered little attention [1]. Yet, the revelation of its topological properties in 2019 [2–6] sparked a surge of research into the MnBiTe family. These extensive studies are primarily driven by two objectives: the realization of a high-temperature quantum anomalous Hall effect (QAHE) and the exploration of exotic phases born from the interplay of topological and magnetic orders, such as the axion insulator phase. Prior to the discovery of MnBi_2_Te_4_, QAHE had been realized in magnetically doped topological insulators (TIs) [7], but the extremely low temperature required by QAHE greatly limited its practical applications. In these magnetically doped TIs, magnetic moments are provided by the dopants. However, the exchange gap is very small, and thus can only survive at extremely low temperatures. Intrinsic magnetic TIs have long been desired to overcome these limitations. Additionally, the theoretical concepts of antiferromagnetic TIs and axion insulators have long been established [8,9], awaiting the discovery of materials capable of embodying these properties.

The MnBiTe family, represented by MnBi_2_Te_4_, serves as an ideal platform to explore the interplay between topological and magnetic orders. Characterized by the layered crystal structure inherent to the MnBiTe family, the magnetic moments of the Mn layers align in a ferromagnetic order along the out-of-plane direction. Depending on the specific stacking of the MnBiTe compound, these Mn layers may adopt either ferromagnetic or antiferromagnetic ordering with adjacent Mn layers. This versatile magnetic behavior, coupled with the system's topological characteristics, makes the MnBiTe family a focus of cutting-edge research in quantum materials. This special topic features four comprehensive reviews and three original research articles, offering insightful perspectives and the most recent advancements in the field.

The first review [10], authored by Shuai Li *et al.* at Southern University of Science and Technology (SUSTech), Tsinghua University and Peking University, provides a comprehensive overview of the advancements in MnBi_2_Te_4_, specifically detailing the crystal, magnetic and band structures, as well as the properties of thin films and the corresponding transport phenomena. The second review [11], a collaborative effort by the teams of Yue Zhao, Chang Liu, Qihang Liu and Chaoyu Chen at SUSTech, delves into the energy gap behavior of the surface state in the MnBiTe family. The third review [12], contributed by Chaowei Hu, Tiema Qian and Ni Ni from the University of California, Los Angeles (UCLA), focuses on single-crystal growth, characterization of chemical disorder and manipulation of magnetism through chemical substitution and external pressure within the same material family. Lastly, the final review [13], penned by Runzhe Xu, Lixuan Xu and Lexian Yang from Tsinghua University, alongside Zhongkai Liu and Yulin Chen from ShanghaiTech University, examines the band structure of MnBi_2_Te_4_/(Bi_2_Te_3_)_*n*_ at different temperatures, doping levels and film thicknesses, as measured by angle-resolved photoemission (ARPES).

In the accompanying three research papers, Wan *et al.* [14] theoretically propose that axion insulators with a simple ferromagnetic configuration, such as the MnBi_2_Te_4_/(Bi_2_Te_3_)_*n*_ family, serve as an ideal setting for exploring the topological magnetoelectric effect. Chen *et al.* [15] uncover a universal origin of the experimentally reported layer Hall effect in MnBi_2_Te_4_ thin films [16] in terms of the hidden Berry curvature, and provide material design principles. Lastly, Bai *et al.* [17] demonstrate the quantized anomalous Hall resistivity in molecular beam epitaxy (MBE)-grown MnBi_2_Te_4_ thin films, noting that elongated post-annealing significantly elevates the temperature required for quantization of the Hall resistivity, albeit with an increase in residual longitudinal resistivity.

The articles presented in this special topic highlight the exotic phases and transport phenomena embodied in the MnBiTe family as well as the topical subjects within this field. With rapid advancements being made, the demand for new theories and technologies is growing. It is hoped that this special topic can deepen the understanding of the interplay between topological and magnetic orders, lay the foundation for further research, and promote the application of magnetic topological materials.
